# Tooth Discoloration Induced by Endodontic Sealers of Different Chemical Bases: A Systematic Review

**DOI:** 10.1590/0103-6440202406021

**Published:** 2024-10-28

**Authors:** Julia Menezes Savaris, Luiz Fernando Monteiro Czornobay, Maria Eduarda Paz Dotto, Pablo Silveira Santos, Lucas da Fonseca Roberti Garcia, Filipe Colombo Vitali, Cleonice da Silveira Teixeira

**Affiliations:** 1 Department of Dentistry, Federal University of Santa Catarina, Florianopolis, Santa Catarina, Brazil

**Keywords:** color science, tooth discoloration, endodontics, endodontic sealers, root canal treatment

## Abstract

The present study aimed to assess the tooth discoloration induced by endodontic sealers and establish a correlation between their distinct chemical compositions and this aesthetic concern. Five electronic databases and grey literature were systematically screened to identify studies comparing the tooth discoloration potential of endodontic sealers with different chemical bases. Studies that did not evaluate human teeth or did not employ spectrophotometry as a method for color measurement were excluded. The risk of bias in the included studies was assessed using a modified version of the JBI Critical Appraisal Checklist for Experimental Studies. Data were grouped according to the chemical composition of the sealers and analyzed qualitatively. Fourteen studies were included. None of the studies met all domains of the risk of bias checklist. Qualitative synthesis suggested that zinc oxide and eugenol-based sealers have a greater potential for tooth discoloration. Silicone-based and calcium hydroxide-based sealers demonstrated less potential for tooth discoloration than resin-based sealers and salicylate resin-based sealers containing calcium silicate. All investigated endodontic sealers induced tooth discoloration, which is chemical and time dependent. Zinc oxide and eugenol-based sealers exhibited a greater potential for tooth discoloration, whereas silicone and calcium hydroxide-based sealers showed less potential.

## Resumo

O presente estudo teve como objetivo avaliar a descoloração dentária induzida por cimentos endodônticos e estabelecer uma correlação entre suas distintas composições químicas e essa preocupação estética. Cinco bases de dados eletrônicas e literatura cinzenta foram sistematicamente examinadas para identificar estudos comparando o potencial de alteração de cor dental de cimentos endodônticos de diferentes bases químicas. Estudos que não avaliaram dentes humanos ou não empregaram espectrofotometria como método de medição de cor foram excluídos. O risco de viés dos estudos foi avaliado usando uma versão modificada do checklist de Avaliação Crítica da JBI para Estudos Experimentais. Os dados foram agrupados de acordo com a composição química dos cimentos e analisados qualitativamente. Quatorze estudos foram incluídos. Nenhum dos estudos atendeu a todos os domínios do checklist de verificação do risco de viés. A síntese qualitativa sugeriu que os cimentos à base de óxido de zinco e eugenol têm maior potencial para alteração de cor dental. Os cimentos à base de silicone e à base de hidróxido de cálcio demonstraram menos potencial para alteração de cor dental do que o cimento à base de resina e o cimento à base de resina salicilato contendo silicato de cálcio. Todos os cimentos endodônticos investigados induziram alteração de cor dental, que é dependente do tempo e da composição química. Os cimentos à base de óxido de zinco e eugenol exibiram maior potencial para alteração de cor dental, enquanto os cimentos à base de silicone e hidróxido de cálcio mostraram menos potencial.

## Introduction

Tooth discoloration represents a significant aesthetic concern, often leading to patient dissatisfaction, emotional distress, a lack of confidence, and impaired aesthetics ^1^. In many cases, corrective interventions such as prosthetic restorations or tooth bleaching become necessary ^2^. Tooth discoloration following root canal treatment occurs in over 30% of cases, with the type of endodontic sealer used being a potential contributing factor ^3^ ^,^ ^4^ ^,^ ^5^ ^,^ ^6^ ^,^ ^7^. The primary purpose of obturation is to establish a three-dimensional seal within the root canal system, thereby preventing the ingress of microorganisms and toxins and avoiding coronal and apical leakage ^8^ ^,^ ^9^. This goal is achieved by combining gutta-percha cones with a sealer ^10^. Endodontic sealers are materials used in a pliable state and may consist of various chemical components, which typically dictate their biological and physicochemical properties ^11^.

The extent of tooth discoloration is frequently associated with the chemical composition endodontic sealers ^3^ ^,^ ^4^ ^,^ ^5^ ^,^ ^6^ ^,^ ^7^ ^,^ ^12^ ^,^ ^13^ ^,^ ^14^. Certain chemical components of these sealers can interact with each other and with the dentin substrate, causing a progressive pigmentation of the tooth ^12^ ^,^ ^13^. The color change can begin within a week after the endodontic sealer meets the dentin and may continue progressively for up to three years ^7^ ^,^ ^15^. Additionally, specific components undergo chemical transformations over time, contributing to tooth discoloration ^13^. The displacement and movement of particles and pigments from the sealer toward dental structures produce tooth hues that vary from shades of gray and brown to yellowish ^13^ ^,^ ^16^.

Although primary studies have investigated the tooth discoloration potential of different endodontic sealers ^3,^ ^4^ ^,^ ^5^ ^,^ ^6^ ^,)^, evaluating these studies individually often provides a limited perspective on the available evidence. To our knowledge, no previous systematic review has synthesized studies on this topic or assessed their methodological quality. By pooling, analyzing, and evaluating data from multiple studies, it can provide more comprehensive and reliable evidence for clinical practice. Furthermore, given the considerable variety of endodontic sealers available, clinicians must be aware of the tooth discoloration potential associated with each type of sealer ^17^. Hence, the present systematic review aimed to evaluate the extent of tooth discoloration induced by various endodontic sealers and establish correlations between their distinct chemical compositions and this clinical concern.

### Materials and Methods

### Protocol and registration

This review is reported according to the Preferred Reporting Items for Systematic Reviews and Meta-Analyses (PRISMA) statement ^18^. A protocol following the PRISMA 2020 statement was previously registered at the International Prospective Register of Systematic Reviews (PROSPERO) platform (CRD42023437956) ^19^.

### Eligibility criteria

The acronym PECOS (Participants, Exposition, Control, Outcomes, and Study Design) was used to determine the eligibility criteria, as follows: (P) human permanent teeth undergoing non-surgical endodontic treatment; (E) use of endodontic sealers of different chemical bases for obturation; (C) tooth crown color before endodontic treatment; (O) tooth crown color after endodontic treatment; (S) laboratory-based studies. Therefore, laboratory studies that compared tooth crown color changes after endodontic treatment using different endodontic sealers for obturation were included. Studies involving immature or animal teeth or those not assessing tooth crown discoloration using a spectrophotometer were excluded. No time or language restrictions were applied.

### Information sources and search strategy

A search strategy was developed using potentially relevant terms identified in controlled vocabularies (DeCS, MeSH, and Emtree terms) and through reading indexed articles on the topic (free terms). The strategy was adapted according to the specificities of each database/repository selected for the search (Supplementary Box 1).

The authors conducted the search on June 26^th^, 2023, and updated it on January 15^th^, 2024. The databases screened include Embase, Latin American and Caribbean Health Sciences (LILACS), PubMed, Scopus, and Web of Science. Additionally, a comprehensive exploration of grey literature was conducted through Google Scholar and ProQuest dissertations & theses. To ensure a broad retrieval of the literature, the reference lists of included studies were checked, and experts were contacted via email, through two attempts at 7-day intervals. The EndNote X7 (Thomson Reuters, Philadelphia, PA, USA) software was used to manage references and eliminate duplicates.

### Study selection

The study selection process for this systematic review was based on a two-phase approach, with two independent reviewers (J.M.S. and L.F.M.C.) First, the reviewers performed an initial screening of the studies by reading their titles and abstracts using a systematic review web application (Rayyan, Qatar Computing Research Institute, Al-Rayyan, Qatar) ^20^. Subsequently, only the studies that met the eligibility criteria were evaluated in detail by full-text reading. In cases where disagreements could not be resolved by the reviewers after discussion and consensus, a third reviewer (M.E.P.D) was consulted for the final decision.

### Data collection process

Two independent reviewers (J.M.S. and L.F.M.C.) conducted data extraction from the included studies using a specific form. Once selected, a third reviewer (M.E.P.D) revised all collected data. Data extraction included publication details (authors, year, and country), sample data (number and type of teeth evaluated), methodological details (groups [n], type of endodontic sealer and obturation technique), outcome assessment method (colorimetric reading method and time, changes in tooth color ((E) [mean and standard deviation]), main findings, funding sources and conflict of interest. In case of absence or incomplete data, the corresponding author of the study was contacted via email for clarification.

**Box 1:**
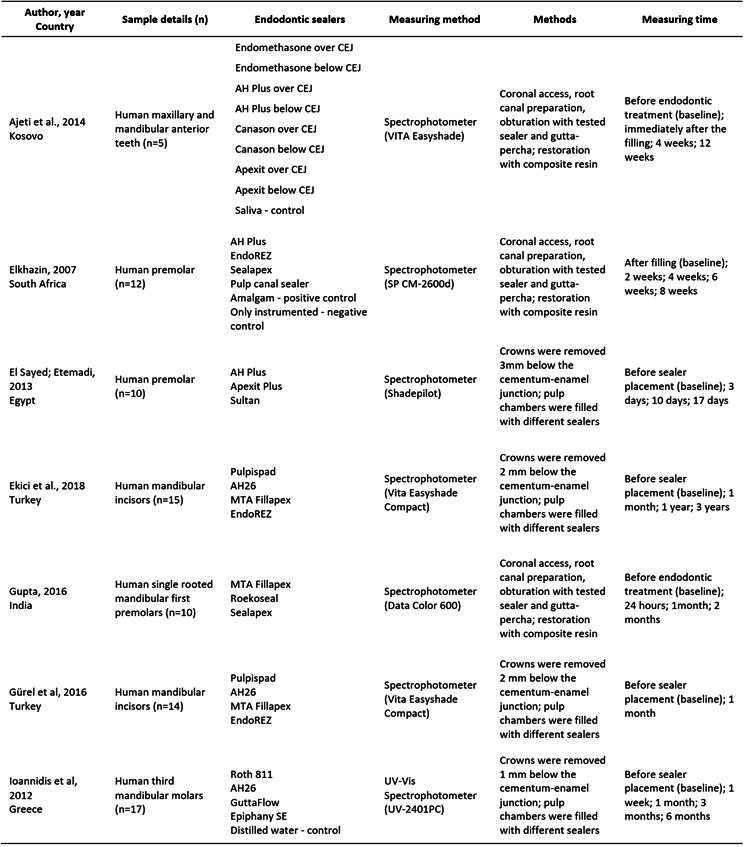
Main characteristics of the studies included in the systematic review.

**Box:**
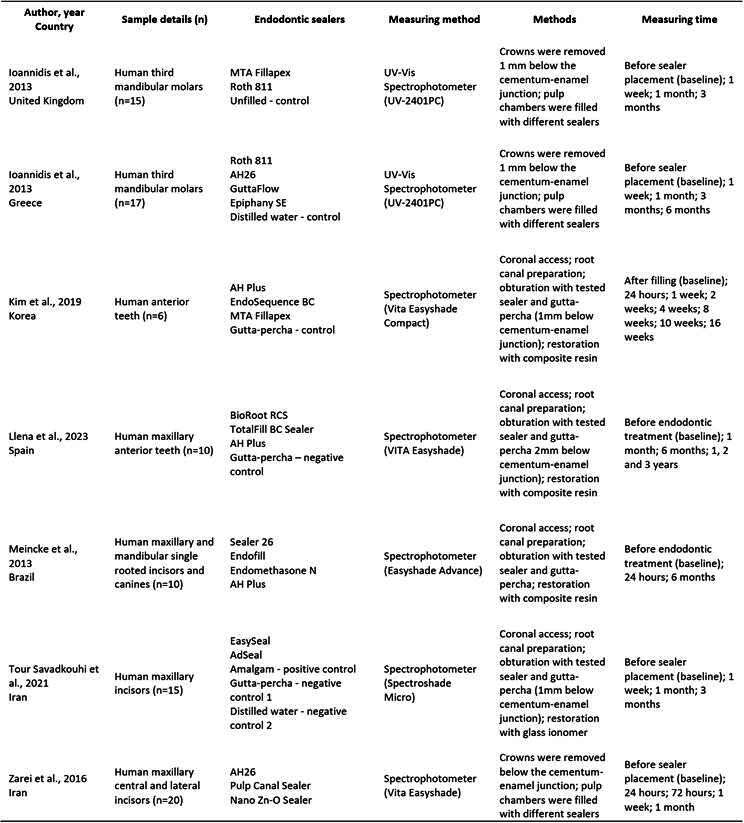
1. Continuation

### Risk of bias assessment

The risk of bias in the included studies for this systematic review was assessed using a modified version of the Joanna Briggs Institute’s (JBI) Critical Appraisal Checklist for Experimental Studies ^21^ ^,^ ^22^. The following items were evaluated: clearly stated aim, sample size justification, sample randomization, equivalence of control and intervention groups at baseline, possibility of comparison between control and intervention groups, clearly described interventions, measurement standardization, reliable measurement methods, and appropriate statistical analysis. Two reviewers (J.M.S. and L.F.M.C.) were previously trained to use this checklist, discussing and standardizing the criteria to be evaluated within each item. Afterward, the same reviewers assessed each item on a three-point scale: ‘yes’, ‘no’, or ‘unclear’. If disagreements arose, they were discussed and judged. In case of no consensus, the third reviewer (M.E.P.D.) was consulted for the final decision.

### Synthesis methods

Initially, the plan was to analyze the data using statistical methods to compare the tooth discoloration potential of different endodontic sealers. However, after a comprehensive examination of the included studies, conducting a meta-analysis was deemed impractical due to substantial methodological differences among them ^23^. Therefore, the data were synthesized according to the chemical bases of the endodontic sealers and reported narratively, following the Synthesis Without Meta-analysis (SWiM) guideline ^24^.

## Results

### Studies selected and their main characteristics

**Figure 1:**
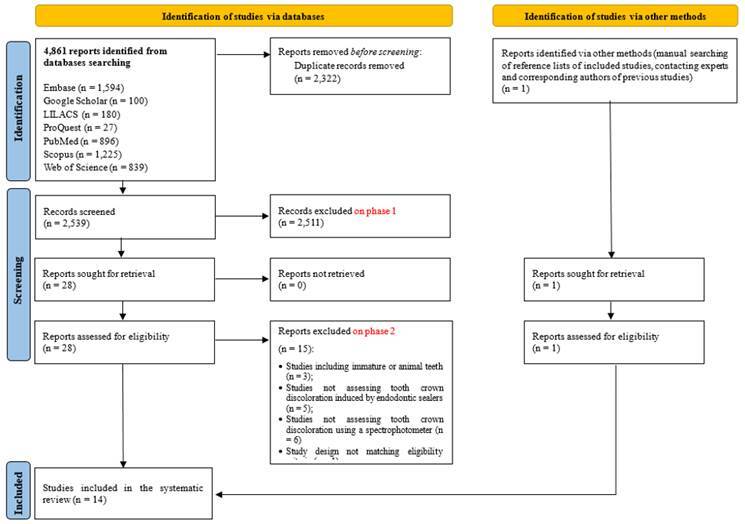
Preferred Reporting Items for Systematic Reviews and Meta-Analyses flow chart showing the selection process.

A total of 4,861 references were retrieved through the searches. After removing duplicates, 2,539 studies were screened by titles and abstracts reading. Following this phase, 28 studies were evaluated in detail through full-text reading (Supplementary Table 1). At the end of the selection process, 14 studies were included in this systematic review. The study selection process is summarized in Figure 1.

**Table:** 1. Risk of bias assessment of studies included.

Quality criteria	Was the aim of the study clearly stated?	Was the sample size justified?	Was the assignment to treatment groups truly random?	Were control and treatment groups comparable at entry?	Were groups treated identically other than for the named interventions?	Were treatments/intervention protocols clearly described?	Were outcomes measured in the same way for all groups?	Were outcomes measured in a reliable way?	Was appropriate statistical analysis used?
Ajeti et al. (2014)	Y	N	U	Y	Y	Y	Y	Y	U
Elkhazin (2007)	Y	N	U	Y	Y	Y	Y	Y	U
El Sayed et al. (2013)	Y	N	U	Y	Y	Y	Y	Y	U
Ekici et al. (2013)	Y	N	U	Y	Y	Y	Y	Y	U
Gupta et al. (2016)	Y	N	U	Y	Y	Y	Y	Y	U
Gurel et al. (2016)	Y	N	U	Y	Y	Y	Y	Y	U
Ioannidis et al. (2012)	Y	Y	U	Y	Y	Y	Y	Y	U
Ioannidis et al. (2013a)	Y	Y	U	Y	Y	Y	Y	Y	U
Ioannidis et al. (2013b)	Y	Y	U	Y	Y	Y	Y	Y	Y
Kim et al. (2019)	Y	N	U	Y	Y	Y	Y	Y	U
Llena et al. (2023)	Y	N	U	Y	Y	Y	Y	Y	U
Meincke et al. (2013)	Y	N	U	Y	Y	Y	Y	Y	U
Savadkouhi et al. (2021)	Y	Y	U	Y	Y	Y	Y	Y	U
Zarei et al. (2016)	Y	N	U	Y	Y	Y	Y	Y	U

Abbreviations: (Y) Yes, (N) No, (U) Unclear

Tool: Modified version of the Joanna Briggs Institute’s (JBI) Critical Evaluation Checklist for Experimental Studies

Box 1 presents the main characteristics of the included studies. All studies were laboratory-based and conducted in various countries: Brazil ^4^, Egypt ^25^, Greece ^14^ ^,^ ^26^, India ^27^, Iran ^6^ ^,^ ^28^, Korea ^29^, Kosovo ^5^, Spain ^15^, South Africa ^30^, Turkey ^7^ ^,^ ^31^, and the United Kingdom ^3^. The studies were published between 2007 and 2023.

Tooth crown discoloration induced by the endodontic sealers was recorded according to the International Commission on Illumination (CIE) L*a*b* system. The tooth crown discoloration over time, denoted as “color change” (ΔE), was determined by analyzing the L*a*b* coordinates to compare the sealers at different time points (Supplementary Table 3). A total of 745 human teeth were assessed for tooth crown discoloration using various types of spectrophotometers. Among these, 53.70% were anterior teeth, 12.76% were premolars, and 27.52% were third molars.

A total of 22 distinct commercial brands of endodontic sealers were included in the evaluation. Among these, 7 exhibited variations in their chemical bases, as follows: ZOE-based sealers (Canason, Endofill, Endomethasone, Pulp Canal Sealer, Pulpispad, Roth 811, and Sultan), resin-based sealers (AdSeal, AH Plus, AH-26, EasySeal, EndoREZ, Epiphany SE, and Sealer 26), a salicylate resin-based sealer containing calcium silicate (MTA Fillapex), calcium silicate-based sealers (EndoSequence BC, BioRoot RCS, TotalFill BC Sealer), silicone-based sealers (GuttaFlow and Roekoseal), a calcium hydroxide-based sealer (Apexit) and a polymeric calcium hydroxide-based sealer (Sealapex). Supplementary Table 4 displays the detailed composition of each sealer tested.

The period of analysis for tooth color change in the included studies varied. The baseline (initial color reading) was generally established before the placement of the sealer into the root canal, as indicated in eleven studies ^3^ ^,^ ^4^ ^,^ ^5^ ^,^ ^6^ ^,^ ^7^ ^,^ ^14^ ^,^ ^15^ ^,^ ^25^ ^,^ ^26^ ^,^ ^27^ ^,^ ^28^ ^,^ ^31^. Only two studies established the baseline upon completion of root canal obturation ^29^ ^,^ ^30^. Measurement periods varied among included studies, ranging from 24 hours to 3 years.

### Risk of bias

Table 1 summarizes the risk of bias assessment. None of the included studies met all domains of the assessment tool, suggesting potential bias in their design, conduct, or data analysis. Methodological concerns were identified in only three domains: domain 2 (71.42% did not perform a proper sample size calculation), domain 3 (none of the studies clearly reported whether sample randomization was performed), and domain 9 (92.85% did not clearly describe the statistical method).

## Synthesis of the results

### Overall analysis

The qualitative synthesis of the included studies revealed that all endodontic sealers, regardless of chemical composition or commercial brand, caused some degree of tooth discoloration after endodontic treatment over time. Detailed information about the performance of different chemical bases is provided below.

### ZOE-based sealers

Ten studies evaluated the tooth discoloration potential of ZOE-based sealers ^3^ ^,^ ^4^ ^,^ ^5^ ^,^ ^6^ ^,,^ ^14^ ^,^ ^25^ ^,^ ^26^ ^,^ ^31^. Overall, this type of sealer induced the most significant tooth discoloration when compared to endodontic sealers of other chemical bases. Two studies ^(^ ^14^ ^,^ ^26^ reported that, after one week, the Roth 811 sealer (ZOE-based) caused severe tooth discoloration. After 1 month, Pulpispad sealer (ZOE-base) affect significantly the tooth color ^31^. After three years of observation, Ekici et al. ^7^ also reported that Pulpispad sealer caused more intense discoloration compared to resin-based sealer (AH26) and salicylate resin-based sealer containing calcium silicate (MTA Fillapex). Furthermore, Endomethasone (ZOE-based) also showed progressive discoloration over 12 weeks ^5^ and 6 months ^4^.

In contrast, two studies ^6^ ^,^ ^30^, which compared the ZOE-based sealer (Pulp Canal Sealer) over 1 month ^6^ and 2 months ^30^ with resin-based and calcium hydroxide-based sealers, did not observe any differences in the degree of tooth discoloration caused by the sealers.

### Resin-based sealers

Twelve studies assessed resin-based sealers ^3^ ^,^ ^4^ ^,^ ^5^ ^,^ ^6^ ^,^ ^7^ ^,^ ^15^ ^,^ ^25^ ^,^ ^26^ ^,^ ^28^ ^,^ ^29^ ^,^ ^30^ ^,^ ^31^. Two studies ^3^ ^,^ ^26^ indicated that AH-26 (epoxy-resin-based sealer) caused more discoloration compared to a silicon-based (GuttaFlow) and methacrylate-resin-based (Epiphany) sealers. After 1 month, when compared with two other sealers (MTA Fillapex and EndoREZ), the teeth filled with the AH-26 showed the greatest crown pigmentation ^7^. However, after 3 years, this pigmentation equalized with the other sealers (MTA and EndoREZ) ^7^. Similarly, AH-26 caused more pigmentation in 1 month ^31^ and 6 months ^4^ compared to a ZOE-based sealer. About AH Plus (epoxy-resin-based sealer), one study ^15^ demonstrated that, after six months, the maximum darkening degree was observed for the resin-based sealer. Also, another study demonstrated that the color change caused by AH Plus was more intense than that caused by the calcium hydroxide-based sealer (Apexit) ^25^.

In four studies ^5^ ^,^ ^28^ ^,^ ^29^ ^,^ ^30^, the resin-based sealers were statistically equivalent to the other types of sealers, regardless of their chemical composition.

### Salicylate resin-based sealer containing calcium silicate

The discoloration potential of a salicylate resin-based sealer containing calcium silicate (MTA Fillapex) was assessed in five studies ^7^ ^,^ ^14^ ^,^ ^27^ ^,^ ^29^ ^,^ ^31^. When compared to a silicon-based (Roekoseal) and a polymeric calcium-hydroxide-based (Sealapex) sealers, MTA Fillapex induced greater tooth discoloration, in a time 2-month observation time ^27^. However, compared to Roth 811 (ZOE-based), MTA Fillapex caused less tooth discoloration ^14^. Over 1 month ^7^ ^,^ ^31^ to 1 year ^7^, MTA Fillapex induced less tooth pigmentation compared to the epoxy-resin-based sealer (AH-26). In comparison with an epoxy-resin-based (AH Plus) and a calcium silicate-based (EndoSequence BC) sealers, MTA Fillapex exhibited similar tooth discoloration over time ^30^.

### Calcium silicate-based sealers

The influence in tooth color alteration of calcium silicate-based sealers (Endosequence BC, BioRoot RCS, and TotalFill BC) was verified in two studies ^15^ ^,^ ^29^. The Endosequence BC sealer, in an observation time of 16 weeks, did not show significant differences in tooth pigmentation compared to other sealers (AH Plus and MTA Fillapex) ^29^. After three years, calcium silicate base sealers (BioRoot RCS and TotalFill BC) exhibited levels of discoloration within the acceptable range, with lower (E values compared to AH Plus sealer ^15^.

### Calcium hydroxide-based sealers

Two studies investigated a calcium hydroxide-based sealer (Apexit) ^5^ ^,^ ^25^, comparing it to AH Plus (epoxy-resin-based), Sultan, Endomethasone, and Canason sealers (ZOE-based). In all these cases, Apexit sealer induced lesser tooth discoloration within a timeframe of 17 days to 12 weeks.

## Discussion

Studies have consistently highlighted a significant correlation between the degree of tooth discoloration and the chemical composition of endodontic sealers ^31^ ^,^ ^32^ ^,^ ^33^. Therefore, the primary objective of this systematic review was to compare the potential for tooth discoloration among several types of endodontic sealers and to explore the relationship between their chemical composition and this clinical concern. The qualitative synthesis underscored that ZOE-based sealers exhibited a higher propensity for tooth discoloration compared to sealers of other chemical bases. Conversely, silicone and calcium hydroxide-based sealers were associated with less tooth discoloration compared to resin and calcium silicate-based sealers.

Several factors contribute to tooth discoloration following root canal treatment, including the persistence of necrotic pulp tissue or filling material remnants in the pulp chamber, and the usage of specific intracanal dressings and irrigation solutions ^33^. A common clinical challenge is the presence of residual filling material, particularly sealer, in the pulp chamber, which can darken over time ^33^. Furthermore, the infiltration of endodontic sealer particles into the dentinal tubules also plays a role in tooth discoloration ^34^ ^,^ ^35^. This phenomenon arises from the corrosion of heavy metal additives, such as silver, leading to the formation of metallic oxides ^13^. A previous study reported three clinical cases indicating that endodontic sealers containing bismuth oxide, when used in conjunction with high-power light-curing, are likely responsible for the delayed darkening of non-vital teeth following the cementation of ceramic veneers ^36^. Therefore, the chemical composition of the endodontic sealer directly impacts the degree of tooth discoloration post-root canal treatment.

All studies included in this systematic review evaluated tooth discoloration in permanent human teeth using spectrophotometers. Spectrophotometry stands out as one of the most precise methods for quantifying color changes on teeth or dental materials over time, and it is widely adopted in laboratory-based studies ^37^ ^,^ ^38^. This approach enhances measurement accuracy through light reflectance, ensuring the reliability, precision, and repeatability of the collected data ^39^. Tooth discoloration was measured in all studies using the CIE L*a*b* formula, which constitutes a three-dimensional and uniform color space system. This system incorporates L* (lightness), a* (red to green), and b* (yellow to blue) coordinates as specific parameters to calculate color changes over time ^40^. Object interaction is numerically expressed through their Euclidean distance, measured as ΔE values at various time points ^39^. However, most current investigations assessing color changes have adopted the CIEDE2000 formula.

The CIEDE2000 formula offers a more precise adjustment compared to the CIE L*a*b* for detecting small visual tolerances commonly observed in clinical practice ^40^. Therefore, laboratory-based findings should be evaluated against color difference thresholds related to "perceptibility" and "acceptability" to ensure real-life relevance ^41^. Perceptibility refers to a scenario where observers can detect a color difference between two distinct objects, while acceptability refers to a situation where the observed color difference is considered acceptable ^41^. A multi-center study on color difference tolerances in dentistry made a significant contribution to ISO/TR 28642 Dentistry-Guidance on color measurement in 2015 ^42^. This study provided crucial insights and established a 50:50% criterion for "perceptibility" and "acceptability" under simulated clinical conditions using both the CIE L*a*b* and CIEDE2000 formulas. For CIEDE2000, the reported threshold values were 0.8 ΔE for "perceptibility" and 1.8 ΔE for "acceptability". Another subsequent study ^41^ offered an interpretation of color differences based on a 50:50% approach for "perceptibility" and "acceptability", building upon their previous work ^42^. From this data, the authors proposed a new classification for mismatch types as moderately unacceptable (>1.8 to ≤3.6 ΔE), clearly unacceptable (>3.6 to ≤5.4 ΔE), and extremely unacceptable (>5.4 ΔE) ^41^.

When comparing the results of the studies included in this systematic review with the mismatch classification types proposed ^41^, it was evident that all tested endodontic sealers caused visually noticeable tooth discoloration over time. A ZOE-based sealer (Pulpispad) caused the most significant tooth discoloration (ΔE=26.2) after 3 years of root canal obturation ^7^, which falls into the category of extremely unacceptable according to clinical standards ^41^. Conversely, one study ^25^ reported that a calcium hydroxide-based sealer (Apexit), a resin-based sealer (AH Plus), and a ZOE-based sealer (Sultan) induced tooth discoloration of less than 1.8 (moderately unacceptable) ^38^. However, this assessment was conducted only 72 hours after completion of endodontic treatment, highlighting the time-dependent nature of tooth discoloration. Peaks in the release of chemical components from the sealers during specific periods, followed by stabilization or reduction, significantly influence their aesthetic impact ^43^. Thus, extending the analysis period is crucial for obtaining more reliable results in studies involving color science ^44^.

The qualitative analysis highlighted a notable potential for tooth discoloration among ZOE-based sealers ^3^ ^,^ ^4^ ^,^ ^5^ ^,^ ^6^ ^,^ ^7^ ^,^ ^14^ ^,^ ^25^ ^,^ ^26^ ^,^ ^31^. Zinc oxide is commonly combined with eugenol to create a filling material with a plastic consistency, resulting in a material with a weak chemical bond between these compounds ^14^ ^,^ ^44^. Even after the setting reaction is completed, there is a continuous release of eugenol, which undergoes auto-oxidation, leading to tooth discoloration over time ^13^ ^,^ ^14^ ^,^ ^44^. This phenomenon was evident in the studies included, where the difference in discoloration was significant only between ZOE-based sealers and other types of sealers at follow-up times longer than 6 months ^4^ ^,^ ^7^.

Regarding resin-based sealers, their composition can vary significantly among manufacturers, potentially affecting the discoloration potential of the sealers. As a result, the impact of these sealers on inducing tooth discoloration presents conflicting findings across studies ^7^ ^,^ ^31^. AH-26 is known for its continuous release of silver ions during and after the setting process, which can contribute to discoloration ^3^ ^,^ ^12^ ^,^ ^26^ ^,^ ^46^. In contrast, AH Plus, recognized as a gold standard endodontic sealer, is a silver-free biomaterial that exhibits a lower tendency for tooth discoloration compared to AH-26 ^47^. Specifically, regarding AH Plus, previous investigations have demonstrated excellent color stability for more than six months ^47^ ^,^ ^48^. However, other authors have reported clinically noticeable color changes within 10 days, which intensified over time ^49^.

The findings from the included studies indicated that calcium hydroxide and silicone-based sealers induced less tooth discoloration compared to other types of sealers ^3^ ^,^ ^6^ ^,^ ^25^ ^,^ ^26^ ^,^ ^27^. In two studies ^5^ ^,^ ^25^, the Apexit sealer, which is calcium hydroxide-based, showed the least alteration in tooth color when compared to AH Plus, Sultan, Endomethasone, and Canason. Regrettably, limited data are available regarding the staining potential of calcium hydroxide-based sealers ^50^. However, it appears that the composition of these sealers might have contributed to the results, as they do not contain significant quantities of silver particles or other heavy metals ^13^ ^,^ ^34^. GuttaFlow, a silicone-based sealer, also demonstrated a lower risk of potential discoloration effects compared to Roth 811, AH-26, and Epiphany SE ^3^ ^,^ ^26^. This outcome is likely due to the satisfactory and improved physicochemical properties of the new-generation sealer in terms of its chemical stability ^3^.

The risk of bias for the included studies was assessed using the JBI Critical Appraisal Checklist for Experimental Studies ^22^. None of the studies met all checklist domains, indicating potential methodological limitations. One notable concern was the absence of sample calculation or justification for the sample size. Establishing parameters for sample size is crucial to ensure that the results have statistical power; however, only 28,6% of the studies met this criterion. Another issue was the randomization of specimens. Although the studies included were *in vitro* investigations, it is highly recommended that samples be randomly assigned to groups to achieve a balance of confounding factors at the baseline. Additionally, for preliminary control and uniform distribution of teeth within each experimental group, a prior color measurement must be conducted using a colorimetric device ^44^. This procedure is essential to ensure the grouping of teeth that are as similar as possible from a colorimetric standpoint ^44^. It was also noted that examiners were not blinded during the assessment of results. According to the JBI tool, when it is not feasible to blind the examiners, the random allocation of specimens is crucial to minimize bias. However, none of the included studies clearly reported whether sample randomization was implemented.

The present systematic review has several limitations that warrant discussion. Firstly, significant heterogeneity was observed among the studies included, which can be attributed to variations in sealer application methods, assessment periods, and types of sealers used. This heterogeneity impacted the inability to conduct the meta-analysis. Additionally, the studies included in this review were primarily *in vitro* investigations, necessitating caution when extrapolating the results to clinical practice. Indeed, conducting a systematic review and meta-analysis of clinical studies would be ideal for comprehensively assessing the impact of different endodontic sealers on tooth discoloration in real-world scenarios. Clinical studies would provide valuable insights into the actual effects of endodontic sealers on tooth discoloration in patients, considering factors such as treatment protocols, patient characteristics, and long-term outcomes. However, this type of study is limited to laboratory research. Further research should focus on well-designed clinical studies to validate the findings from laboratory-based investigations and provide more clinically relevant evidence.

It is also important to note that some studies intentionally left sealer in the pulp chamber to simulate a 'worst-case' clinical situation ^14^. However, in clinical practice, it is well-established that obturation materials (sealer and gutta-percha) should be placed below the cervical region to prevent prolonged interaction over time, such as the sealer within the dentin tubules ^16^. A study conducted on bovine incisors found that setting a cervical limit of the filling material 2 mm from the cementum-enamel junction is advisable, as it reduces crown discoloration over a one-year observation period ^51^.

Based on the limitations identified in the studies included in this systematic review, the authors suggest that further studies establish parameters for determining the appropriate sample size for their investigations. Additionally, research should focus on investigating tooth discoloration induced by tricalcium silicate-based sealers in conventional root canal treatment, as limited literature exists on this aspect. There are controversies regarding the tooth discoloration potential of resin and ZOE-based sealers, which are widely used in current clinical practice, and further studies are needed to clarify these controversies. Clinical studies are highly recommended to validate findings from laboratory-based investigations and provide more clinically relevant evidence, as only one cross-sectional study has reported tooth discoloration measures following root canal treatment ^52^. Longitudinal studies that include monitoring patients who have undergone endodontic treatment are especially encouraged, as they can provide more solid evidence regarding the causality of tooth discoloration related to root canal treatment and endodontic sealers of different chemical bases.

In conclusion, this systematic review demonstrated that all investigated endodontic sealers have the potential to cause tooth discoloration. This discoloration is influenced by both the chemical composition of the sealers and time. Notably, ZOE-based sealers showed a higher propensity for tooth discoloration compared to other types of sealers. Moreover, the available evidence indicates that silicone and calcium hydroxide-based sealers tend to induce less tooth discoloration than resin-based and salicylate resin-based sealers containing calcium silicate. It is crucial to highlight that these conclusions are based on *in vitro* studies, and caution should be exercised when applying these findings to clinical practice.

### Deviations from the registered protocol

Inclusion criteria: The original plan was to also include clinical studies. However, upon reviewing the available literature, we found that only in vitro studies were available. Consequently, the inclusion criteria were revised. Data analysis: Initially, a statistical method was designed to compare tooth discoloration data between different groups of endodontic cements. However, after a critical analysis of study methodologies and identification of substantial between-study heterogeneity, it was determined that conducting a meta-analysis was not feasible. Therefore, the analysis was adjusted to a narrative synthesis, descriptively summarizing the findings.
